# Using conjoint analysis to develop a system of scoring policymakers’ use of research in policy and program development

**DOI:** 10.1186/s12961-015-0022-y

**Published:** 2015-08-04

**Authors:** Steve R Makkar, Anna Williamson, Tari Turner, Sally Redman, Jordan Louviere

**Affiliations:** 1 The Sax Institute, Level 13, Building 10, 235 Jones Street, Ultimo, NSW 2007 Australia; 2 World Vision Australia, 1 Vision Drive, Burwood East, Melbourne, Victoria 3151 Australia; 3 School of Marketing, University of South Australia, Level 4, Yungondi Building, North Terrace, Adelaide, South Australia 5000 Australia

**Keywords:** Conjoint analysis, Evidence-based policy, Evidence-informed policy, Health policy, Knowledge translation, Measurement, Policymaker, Research, Use, Utilisation

## Abstract

**Background:**

The importance of utilising the best available research evidence in the development of health policies, services, and programs is increasingly recognised, yet few standardised systems for quantifying policymakers’ research use are available. We developed a comprehensive measurement and scoring tool that assesses four domains of research use (i.e. instrumental, conceptual, tactical, and imposed). The scoring tool breaks down each domain into its key subactions like a checklist. Our aim was to develop a tool that assigned appropriate scores to each subaction based on its relative importance to undertaking evidence-informed health policymaking. In order to establish the relative importance of each research use subaction and generate this scoring system, we conducted conjoint analysis with a sample of knowledge translation experts.

**Methods:**

Fifty-four experts were recruited to undertake four choice surveys. Respondents were shown combinations of research use subactions called profiles, and rated on a 1 to 9 scale whether each profile represented a limited (1–3), moderate (4–6), or extensive (7–9) example of research use. Generalised Estimating Equations were used to analyse respondents’ choice data, which calculated a utility coefficient for each subaction. A large utility coefficient indicated that a subaction was particularly influential in guiding experts’ ratings of extensive research use.

**Results:**

Utility coefficients were calculated for each subaction, which became the points assigned to the subactions in the scoring system. The following subactions yielded the largest utilities and were regarded as the most important components of each research use domain: using research to directly influence the core of the policy decision; using research to inform alternative perspectives to deal with the policy issue; using research to persuade targeted stakeholders to support a predetermined decision; and using research because it was a mandated requirement by the policymaker’s organisation.

**Conclusions:**

We have generated an empirically derived and context-sensitive means of measuring and scoring the extent to which policymakers used research to inform the development of a policy document. The scoring system can be used by organisations to not only quantify the extent of their research use, but also to provide them with insights into potential strategies to improve subsequent research use.

**Electronic supplementary material:**

The online version of this article (doi:10.1186/s12961-015-0022-y) contains supplementary material, which is available to authorized users.

## Background

Research is widely regarded as providing the most reliable evidence source upon which to base decisions relating to health policies, programs, and other courses of action [1-5]. The process of incorporating the best available research evidence to inform decision making relating to programs and public health policies is called evidence-informed health policymaking [6,7]. Evidence-informed health policymaking is purported to lead to better health policies, more effective implementation, and more efficient use of resources, with the ultimate goal of improving health outcomes for the wider community [8,9]. The public health literature describes numerous policies that have been informed by research in a range of health areas (e.g. smoking, alcohol use, immunisation, fall prevention, cardiovascular health, neural development, and mental health [10-16]). Many of these policies have been associated with improvements in health, suggesting a possible link between evidence-informed policymaking and better health outcomes. In light of these potential benefits, policymakers and organisations are showing greater appreciation of the importance and usefulness of research as a source of information to guide decision making [17].

Despite these gains, there are global calls to strengthen the capacity of policy organisations to utilise the best available evidence from research in healthcare practices and policies [18]. This is because, internationally, many opportunities to use research to inform policymaking are missed [17,19-23], ineffective health policies which are not supported by the available evidence continue to be implemented (e.g. [10,24,25]), and healthcare expenditure continues to rise rapidly [26,27].

### The importance of measuring research use in policy and existing measures

In this context, it is essential that validated measures of research use are developed. Such measures will greatly assist policymaking organisations to evaluate their current level of research use, use these findings as a start off point to invest in resources or programs to increase their research use capacity, and monitor the effectiveness of these interventions [28]. Organisations could also use such measures to quantify the impact of research use on health outcomes and financial expenditures. This may motivate ongoing research use by organisational staff, promote funding and production of policy-relevant research by research organisations [8,9,29], and justify continued government investment into research with real-world impacts [30].

Despite these potential benefits, few measures of research use in health policy are available [19,31-37]. The measures that are available have some key limitations such as a lack of a clear theoretical basis, narrow definition of the concept of research use, application to health care as opposed to policy, reliance on self-report, and absence of a valid scoring system [37]. In addition, currently available measures do not assess research use in relation to specific policy products that were recently developed, but instead ask policymakers about their research use in general or over extended periods of time (e.g. 5 years [19]). This lack of time specificity, context, and reference to a concrete document, may lead to difficulties with recall and inaccurate reporting of research use [38,39].

Zardo and Collie [37] developed a content analysis approach to measuring research use, where individual policy documents are coded for the type of evidence cited and how that evidence was used (i.e. to support policy development or guide decision making). The primary limitation with their approach is that only instrumental use and direct references to research are assessed. Consequently, their measure does not take into account uncited research, research that contributed to ideas and concepts surrounding the policy document’s development (i.e. conceptual use [40]), or research that was used to persuade stakeholders or justify predetermined decisions (i.e. tactical use [41]).

### SAGE: A new measure of research use

To overcome the limitations of previous measures, we developed a comprehensive, multi-modal (i.e. interview, document analysis), and theory-based measure of policymakers’ use of research in the development of a recently approved health policy or program document, entitled Staff Assessment of enGagement with Evidence from research (SAGE). Within SAGE, research evidence refers to analyses of quantitative or qualitative data, or theory, found in peer-reviewed articles, books, technical monographs, or grey literature such as internally conducted studies, evaluations, or reports on authoritative websites [42]. SAGE was developed as part of the Centre for Informing Policy in Health with Evidence from Research (CIPHER), which was established with the aim of developing and testing new strategies to increase the use of research evidence in policy, improving policymakers’ access to information summaries, building researchers’ skills in working with policy agencies, and developing new ways of measuring the use of research in policy.

SAGE is informed by the Supporting Policy in Health with Research: an Intervention Trial (SPIRIT) Action Framework, which describes the steps, barriers, facilitators, and contextual influences along the pathway to using research to inform policymaking [28]. The framework provides a simplified schematic to summarise the process through which research informs policymaking, but in no way assumes that policymaking is a linear, predictable process [19]. Specifically, the framework describes that when policymakers seek out research to inform the development of a policy, they initiate a number of research engagement actions (e.g. accessing, appraising, and generating new research). Once relevant research has been obtained and/or generated, it can then be used to inform the key stages of policymaking (e.g. agenda setting, policy development). Research is conceptualised as being used in four different, but non-mutually exclusive ways. Specifically, research may (1) provide new ideas, understanding, or concepts to clarify thinking about the policy issue without directly influencing content (conceptual use [43,44]); (2) directly influence what issues to prioritise and/or what action should be taken to deal with the identified issue(s) (instrumental use [19,45,46]); (3) be used to justify or lend weight to pre-existing decisions and courses of action relating to the issue (tactical use [41,47]); and/or (iv) be used to meet organisational, legislative, or funding requirements to use research (imposed [46]). The Framework predicts that each of the four kinds of research use may lead to more policies that are informed by research and possibly better health services and outcomes, but only if the most relevant and reliable research available is used.

Informed by this framework, SAGE broadly assesses (1) the extent to which policymakers undertook research engagement actions, and (2) the extent to which research was used to inform the development of a policy document. SAGE consists of a comprehensive interview and a scoring tool. In the interview, policymakers are invited to describe how research was searched for, obtained, appraised, generated (i.e. research engagement actions), and used (i.e. research use: conceptual, instrumental, tactical, and imposed) to inform the development of a discrete policy document (see Additional file 1 for the SAGE interview). The interview takes approximately 40 min to complete and is administered by a trained interviewer with experience in qualitative analysis and interviewing.

**Figure 1:**
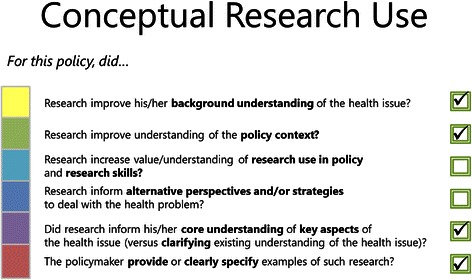
Example scoring checklist for conceptual research use.

The accompanying SAGE scoring tool allows objective assessors to rate the thoroughness with which policymakers undertook research engagement actions and the extent to which research was used in the development of the policy document. In this paper, we describe how the tool was developed to score the research use component of SAGE. The scoring system to assess research engagement actions is described in another paper [48]. The scoring tool is a comprehensive checklist that lists the key subactions of the four research use domains (Figure 1 provides an example of the checklist for tactical use). These subactions are the essential features or actions of each research use domain (see Table 1 for definitions of key terms and examples). For example, subactions of tactical research use include using research to validate a predetermined decision, or using research to persuade stakeholders to support a decision. Using the SAGE interview transcript and the accompanying policy document, external raters mark on the scoring tool whether or not the policymaker undertook each of these key subactions. Such a scoring tool is beneficial in allowing agencies to evaluate policymakers’ current use of research in policy development, as well as the impact of programs designed to improve evidence-informed health policymaking.

**Table 1:** **Definitions of key terms**

Term	Definition	Example
Research use domains	The four ways in which research can be used in policymaking based on the literature on evidence-informed policymaking. Throughout the paper, research use domains are numbered with Arabic numerals.	1. Instrumental use: research directly influences what issues to prioritise and/or what action should be taken to deal with the identified issue(s)
		2. Conceptual use: research is used to provide new ideas, understanding, or concepts to clarify thinking about the policy issue without directly influencing content
		3. Tactical use: research is used to justify or lend weight to pre-existing decisions and courses of action relating to the issue
		4. Imposed use: research is used to meet organisational, legislative, or funding requirements to use research
Subactions	Subactions^a^ are the essential features or main actions of each research use domain. They often refer to broad, concrete example actions of using research in each of the four domains. Each research use domain has a number of subactions that were identified through examination of literature on evidence-informed policymaking and interviews with policymakers. Subactions are numbered with letters.	Examples of subactions of tactical research use include:
		a. Research is used to support, confirm, or justify established positions or decisions relating to the issue
		b. Research is used to provide hard evidence to persuade targeted stakeholders to support an existing decision or view
		c. Research is used to provide hard evidence to persuade peripheral stakeholders to support an existing decision or view
		d. Research is used to inform stakeholders about key issues relating to the health issue
Level	Levels in conjoint analysis refer to all the possible values of a subaction and are often described in concrete terms. To undertake a conjoint analysis, each subaction must be divided into concrete, perceptible levels. In the present study, the majority of subactions were divided into two levels (i) Yes, the subaction was performed by the policymaker, or (ii) No, it was not performed by the policymaker. Different levels of subactions are combined in various combinations to form profiles. Throughout the paper, levels are numbered using Roman numerals.	As above, one of the subactions of tactical research use was “using research to support, confirm, or justify established positions or decisions relating to the issue”. This subaction has two levels:
		(i) Yes, the policymaker used research to support, confirm, or justify an established position or decision relating to the issue
		(ii) No, the policymaker did not use research to support, confirm, or justify an established position or decision relating to the health issue
Profile	A research use profile is made up of a combination of subaction levels. Specifically, a profile consists of one level of each subaction within that research use domain	Using the research use domain – tactical research use, an example profile would be:
		a. (i) Yes, research was used to support, confirm, or justify established positions or decisions relating to the issue
		b. (ii) No, research was not used to provide hard evidence to persuade targeted stakeholders to support an existing decision or view
		c. (ii) No, research was not used to provide hard evidence to persuade peripheral stakeholders to support an existing decision or view
		d. (i) Yes, research was used to inform stakeholders about key issues relating to the health issue

^a^To enhance clarity and comprehension throughout the paper, we used the term subaction instead of attribute, which is most commonly used in choice studies and conjoint analysis.

### Developing a system to score research use

What is missing from the current scoring tool is a system that assigns a numeric score to each subaction and thus enables the calculation of a total score for each research use domain. Assigning an identical score to each subaction is not appropriate given that extensive qualitative research indicates that certain subactions represent stronger examples of a particular research use domain compared to others. For example, using research to persuade stakeholders to support a course of action relating to a health issue [3,47] is a stronger form of tactical research use than using research to inform stakeholders about the issue [49]. Previous research has often involved qualitative interviews with health policymakers and researchers with varying levels of experience and seniority. There has been no attempt, however, to quantify the views of policymakers and researchers regarding the relative importance of different research use subactions. Consequently, an appropriate scoring system cannot be generated on the basis of previous research.

### Using conjoint analysis to develop a system to score research use

One systematic method of quantifying experts’ views regarding the value to assign each research use subaction is conjoint analysis [50-55]. Conjoint analysis has been used in health economics to determine what health products and services patients prefer, and the attributes driving these preferences [52,53,56-59]. In traditional conjoint analysis, respondents rate combinations of subactions^1^ called profiles (see Table 1 for definitions). This is an ecologically valid approach, because each type of research use is composed of several smaller actions [28]. For example, conceptual research use not only includes what knowledge areas were improved by research, but also the extent to which research improved this knowledge. After profiles have been rated, conjoint analysis is used to compute numeric values or utilities for each subaction. These utilities quantify the relative importance of each subaction to each research use domain, based on the opinions and preferences of the chosen sample. These utilities can then be used as the score assigned to each subaction within a scoring tool.

An appropriate sample for the current conjoint analysis would be individuals with extensive experience working at the nexus between health policy and research. Such experts are cognisant of the diverse ways research can influence policy in light of political influences, stakeholder interests, skill and resource limitations, and other contextual factors. Consequently, they can provide informed and context-sensitive judgments regarding the relative importance of each research use subaction, which can then be used to generate appropriate and context-sensitive scores for these subactions.

In summary, the aim of this paper was to use conjoint analysis to generate a context-appropriate and valid system to score policymakers’ research use in policy development, based on the opinions of experts in health policy and research. We envision that the scoring system will help to inform policy organisations of the most important components of research use, which can then be addressed through targeted interventions to build research capacity and use in policymaking.

## Method

### Ethics

Ethics approval was granted by the University of Western Sydney Human Research Ethics Committee HREC Approval H10440. Written consent was obtained from all potential respondents prior to their participation in the study.

### Respondents

In recruiting respondents to undertake the conjoint analysis surveys, we targeted experts working at the nexus between health policy and health research. Firstly, we identified relevant researchers by contacting corresponding authors of key research articles in the area of evidence-informed health policymaking. Secondly, we contacted members of the CIPHER community to identify researchers and policymakers with experience in both health policy and health research. Using this method, 361 experts were invited by email to participate in the study (making them the respondents/participants for the purposes of this paper). Of the invited respondents, 267 (74%) were from Australia, 52 (14%) were from North America (16 United States, 35 Canada, and 1 Mexico), 40 (11%) were from Europe (31 United Kingdom, 1 Austria, 1 Ireland, 1 Sweden, 3 Norway, 3 Netherlands), and 2 (<1%) were from Africa (1 each from Mali and Uganda).

### Procedure

We followed the guidelines specified by Bridges et al. [54] and Lancsar and Louviere [60] for designing, conducting, analysing, and reporting on the findings of choice experiments. Furthermore, we applied principles of Hierarchical Information Integration by separating research use into its key domains (i.e. conceptual, instrumental, tactical, and imposed), identifying key, non-overlapping subactions for each domain, and undertaking a separate choice experiment for each domain [61,62]. These steps are described below.

#### Defining the subactions and levels

We undertook a comprehensive, step-by-step approach to identify the subactions of each research use domain. We first defined each of the four research use domains using the SPIRIT Action Framework [28], seminal research on evidence-informed policymaking, and Haynes and colleagues’ review of health policy definitions [63]. With these definitions in mind, we conducted a thorough analysis of the (1) extant literature on evidence-informed health policymaking, and (2) 65 SAGE interviews with Australian health policymakers from six Australian health organisations, to identify a broad range of concrete examples of each research use domain. Note that these interviewees were not the respondents that completed the conjoint surveys (i.e. they were not the participants in this study).

**Figure 2:**
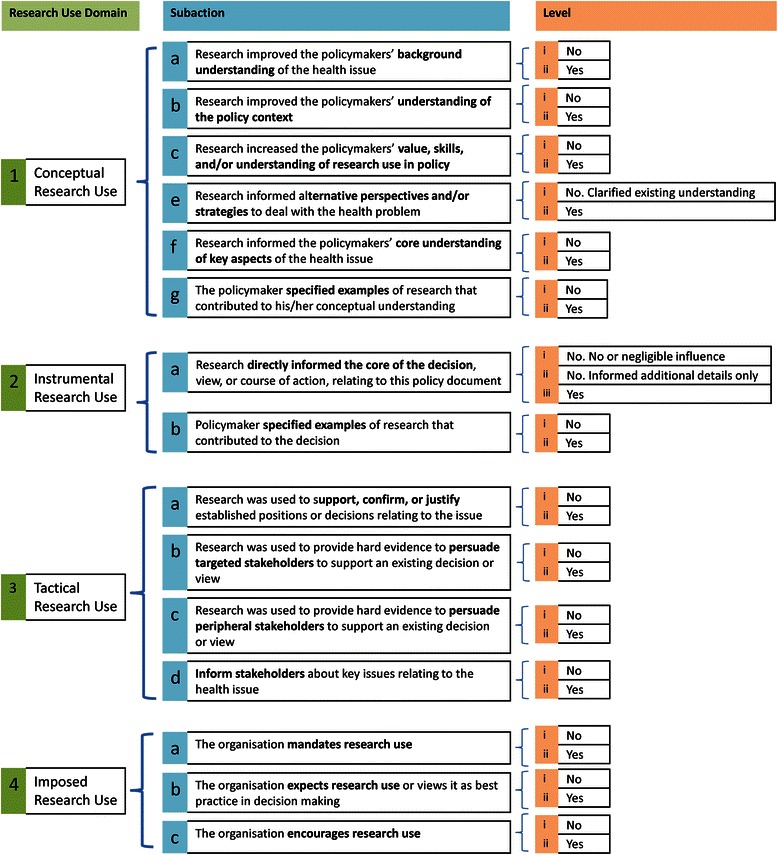
Subactions and levels for each research use domain.

A vast number of examples of each research use domain were identified from the literature (over 100) and interviews (approximately 40). Similar examples were then categorised into groups. Each group was given an action label that encompassed all the examples within that group. These action labels became the subactions for a particular research use domain. For example, using research to understand the current prevalence rate of a disease and using research to understand risk factors for a particular health condition, were both examples of Conceptual Research Use identified in the literature. These two examples were grouped together to form a specific subaction of Conceptual Research Use: using research to inform one’s general background understanding of the health issue (subaction 1a; Figure 2).

Having identified the subactions of each research use domain, the next step involved dividing each subaction into its levels (Table 1). Levels in conjoint analysis refer to the possible values of a subaction [50]. Hair et al. [50] emphasised that levels should be stated in concrete terms. As a result we separated the majority of subactions into just two levels: (i) Yes, the action was performed or (ii) No, the action was not performed. Only one subaction for instrumental use contained three levels, representing the extent to which research influenced the decisions relating to the policy document. Identifying the levels of subactions was a necessary step before conducting the conjoint analysis, so that profiles could be created. Profiles are combinations of subaction levels (Table 1 and Additional file 2). The final list of subactions and their levels for each research use domain is displayed in Figure 2.

#### The experimental design

The full profile method was used [50], where each profile consisted of one level from each subaction within a particular research use domain. The profiles were hypothetical, and presented in relation to policy and program documents in general, rather than in relation to a specific policy document. Therefore, no documents or descriptions of specific policy documents were presented. The subactions and levels gave rise to a large number of possible profiles, particularly for conceptual use. The number of profiles was reduced to a manageable number (i.e. eight profiles for conceptual and tactical, six profiles for instrumental, and four profiles for imposed use) using an Orthogonal Main Effects Plan (OMEP) in R software [64]. The OMEP generated a series of orthogonal and balanced profiles for each of the four conjoint analyses. This was appropriate because we were only interested in main effects (i.e. the utility values assigned to each research use subaction level) rather than interactions among subaction levels [50]. The small number of profiles generated for each research use domain would enhance the efficiency of the task and reduce the cognitive load on our sample. The complete list of profiles for all four research use domains is displayed in Additional file 2.

#### Eliciting preferences

**Figure 3:**
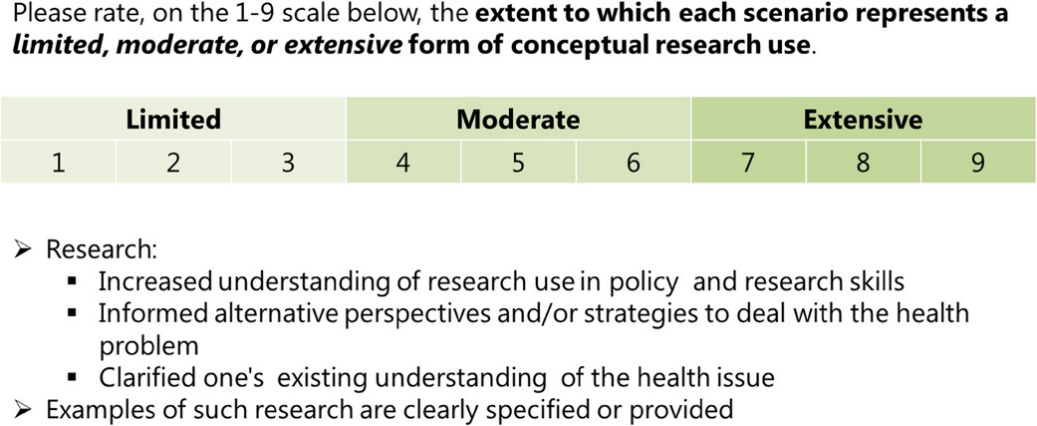
Example scenario for conceptual research use.

In order to elicit respondents’ preferences, they were instructed to rate the standard of each profile on the same 1 to 9 ordinal scale (Figure 3). Profiles were presented using an online survey created using Survey Monkey software [65]. Respondents completed four online surveys, one for each research use domain. The survey order was as follows: Instrumental use, Conceptual use, Tactical use, and Imposed use.

All potential respondents were contacted by email, where study information and a link to the online survey were provided. The first page was an online consent form. On the second page, respondents entered personal details including their assigned ID number (which was sent with their invitation email), affiliation, and current working role, which they could select as either ‘policymaker’, ‘researcher’, ‘both’, or ‘other’. If ‘other’ was chosen, they were required to specify their working role in a textbox. After providing their details, respondents completed the conjoint surveys for research use domain (see [48] for details). Respondents were then presented with the four conjoint surveys for each research use domain. Each survey with its corresponding profiles was presented on a separate page. On each survey page, key terms within profiles were hyperlinked to a glossary page, which opened in a new browser tab when clicked. The glossary provided definitions and concrete examples of all the key terms including the four research use domains and the subactions within each domain in order to assist respondents in making their ratings of each profile. See Figure 3 for an example scenario of conceptual research use that respondents rated. All respondents were presented with the same set of profiles generated from the OMEP. Respondents were required to rate on a 1 to 9 scale, whether the profile represented a limited (1–3), moderate (4–6), or extensive (7–9) form of the research use domain in question. The presentation order of profiles was randomised across respondents. Respondents were required to rate all profiles for a particular research use domain before moving onto the next page.

#### Data analyses

Four conjoint analyses were undertaken using SPSS GENLIN (using a logit link function and robust estimator), which is appropriate when ratings are made on an ordinal scale, predictors are categorical, and participants provide multiple responses [66]. An exchangeable working correlation structure was used because examination of the within-subject correlation matrix revealed that ratings of profiles within subjects were correlated at approximately similar magnitudes [67]. There were problems with convergence due to singularity on one conjoint analysis (i.e. conceptual use). Based on the recommendations of Lipsitz et al. [68], estimates obtained from the first iteration of the GENLIN procedure were used for this research use domain.

Raw regression coefficients for each subaction level were calculated, which represented the part-worth utilities of each subaction level. To make the part-worth utilities meaningful, they were rescaled into a positive value out of 9 using the guidelines provided by Hair et al. [50]. Larger rescaled utility values indicated that a particular subaction (level) was particularly influential in guiding respondents’ ratings. Importance values were calculated using the guidelines of Hair et al. [50] to quantify the relative importance of each subaction. Larger part-worth utilities and importance values indicate that a particular subaction was relatively more influential in guiding respondents’ ratings.

SPSS CONJOINT was used to identify respondents that exhibited reversals; that is, highly inconsistent responses and illogical patterns in preferences for particular subaction levels [50]. Hair et al. [50] proposed that respondents who display many reversals are potential candidates for deletion from the analyses.

## Results

In Table 3, we display the raw and rescaled part-worth utilities for each research use subaction, as well as the importance values for each subaction.

### Respondent characteristics

Out of the 361 participants invited, 54 respondents (14.96%) consented and completed all four surveys. These 54 respondents had earlier completed six other surveys for the research engagement actions component of SAGE [48]. Based on Orme’s [69] guidelines regarding the appropriate sample size for investigational work and developing hypotheses about a particular group (i.e. between 30 and 60), our sample size was sufficient.

Respondent characteristics are displayed in Table 2 for the sample that completed all four surveys (*N* *=* 54). There were significantly more female than male participants (χ^2^(1, *N* = 54) = 6, *P* = 0.01). There were no significant differences relating to the working role of participants (χ^2^(3, *N* = 54) = 1.85, *P* = 0.60), nor was there a significant association between working role and sex (χ^2^(3, *N* = 54) = 2.25, *P* = 0.52). There was a significant effect of geographic region, with most participants coming from Australia, followed by North America (primarily Canada, with one participant from the United States) and then Europe (mainly the UK, with one participant from Norway; χ^2^(2, *N* = 54) = 49.00, *P* <0.001). There was no significant relationship between geographic region and sex (χ^2^(2, *N* = 54) = 5.46, *P* = 0.07).

**Table 2:** **Respondent characteristics**

			Working role					Geographic region (Continent)			
			Policymaker	Researcher	Both researcher and policymaker	Other	Total	Australia	North America	Europe	Total
Sex	Male	Count (% total)	3 (5.6%)	8 (14.8%)	4 (7.4%)	3 (5.6%)	18 (33.3%)	11 (20.4%)	6 (11.1%)	1 (1.9%)	18 (33.3%)
	Female	Count (% total)	10 (18.5%)	9 (16.7%)	10 (18.5%)	7 (13.0%)	36 (66.7%)	31 (57.4%)	3 (5.6%)	2 (3.7%)	36 (66.7)%
Total		Count (% total)	13 (24.1%)	17 (31.5%)	14 (25.9%)	10 (18.5%)	54 (100%)	42 (77.8%)	9 (16.7%)	3 (5.6%)	54 (100%)

### Conjoint analysis findings for each type of research use

#### Conceptual research use

One respondent exhibited six reversals and was eliminated, leaving 53 respondents in the analyses. All raw utility coefficients were highly significant and negative, implying that each subaction was positively associated with greater conceptual use. Based on the rescaled coefficients and importance values, experts’ ratings of conceptual research use were most strongly affected by whether or not the policymaker could specify examples of research that contributed to his/her understanding of the issue (subaction 1g). In terms of the specific type of conceptual understanding (subactions 1a–d), respondents considered conceptual use to be greater when policymakers used research to improve their understanding of alternative strategies to deal with the current health problem (subaction 1d) and the policy context (subaction 1b), relative to improving their background understanding of the issue (subaction 1a) or knowledge and skills in applying research to policy (subaction 1c). Respondents also gave higher ratings if policymakers could describe examples of research that contributed to their increased understanding (subaction 1e). Ratings of conceptual research use were higher if research influenced policymakers’ core, as opposed to their peripheral understanding of the health issue, although this subaction had the lowest relative utility.

#### Instrumental research use

All 54 respondents were included in the analysis. Raw utility coefficients were significant and negative for both subactions, indicating that each was positively associated with greater instrumental research use. Based on the rescaled coefficients, respondents considered instrumental research use to be most extensive if research influenced the core components of the decision or course of action (as opposed to providing additional or vague details) and policymakers could identify the specific research that influenced these decisions. Based on the importance values, the subaction referring to the extent of direct research use within the document (subaction 2a) was two times more important than whether or not the policymaker specified examples of research that influenced the policy (subaction 2b).

#### Tactical research use

All 54 respondents were included in the analysis. Each raw utility coefficient was significant and negative, implying that each of the four subactions was associated with higher tactical use ratings. Based on the rescaled coefficients and importance values, the most important subaction was using research to persuade targeted stakeholders to support or act upon an existing decision (subaction 3b), versus using research to justify a decision (subaction 3a), or inform stakeholders about the health issue (subaction 3d). The latter two subactions had similar importance values. Using research to persuade peripheral stakeholders (subaction 3c) had the lowest rescaled utility, and its importance was almost half that of subaction 3b (i.e. persuading targeted stakeholders).

#### Imposed research use

One respondent exhibited reversals on all subactions and was eliminated, leaving 53 respondents in the analysis. Raw utility coefficients for each subaction were significant and negative indicating that each was associated with more extensive imposed research use. Examining the rescaled coefficients and importance values, respondents’ ratings for imposed use were most strongly influenced by whether organisations mandated research use (subaction 4a) relative to if they expected (subaction 4b) or encouraged research use (subaction 4c). Furthermore, ratings of imposed use were greater if organisations expected (subaction 4b), rather than encouraged (subaction 4c) research use.

#### Using the scoring system

The utilities in Table 3 provided the basis for scoring each research use domain in SAGE. Utilities were rescaled so that they summed to nine within each research use domain. This was done because each research use domain is scored on a scale of 1 to 9 in SAGE (where 1–3 = limited; 4–6 = moderate, and 7–9 = extensive). Thus, the rescaled utility is the score assigned to each research use subaction in the scoring tool. Using policymakers’ responses to the SAGE interview and the accompanying policy document, if a policymaker had engaged in a particular subaction, it is ticked off and the utility score is assigned for that subaction.

**Table 3:** **Research use domains, subactions, subaction levels, raw utilities, standard errors, and rescaled utility coefficients for each domain**

Research use domain	Key subaction	Levels of each subaction	Raw utility coefficient (SE)^a^	Rescaled utility coefficient^b^	Relative importance (%)
1. Conceptual research use	a. Background understanding of the health issue	i. No	−0.92 (0.15)	0	11.47
		ii. Yes	0	1.19	
	b. Understanding of the policy context	i. No	−1.09 (0.14)	0	16.27
		ii. Yes	0	1.41	
	c. Value, skills, and/or understanding of research use in policy	i. No	−0.84 (0.16)	0	9.82
		ii. Yes	0	1.09	
	d. Alternative perspectives and/or strategies	i. No	−1.26 (0.14)	0	18.94
		ii. Yes	0	1.62	
	e. Informed core understanding of the issue	i. No, just clarified	−0.69 (0.12)	0	9.43
		ii. Yes	0	0.88	
	f. Examples were specified	i. No	−2.18 (0.23)	0	34.07
		ii. Yes	0	2.81	
2. Instrumental research use	a. Research informed the core of the decision	No/negligible influence	−4.34 (0.50)	0	69.37
		ii. No, additional details only	−2.37 (0.33)	2.15	
		iii. Yes	0	4.75	
	b. Examples were specified	i. No	−1.92 (0.27)	0	30.63
		ii. Yes	0	2.10	
3. Tactical research use	a. Support, confirm, or justify predetermined decisions	i. No	−1.44 (0.19)	0	24.59
		ii. Yes	0	2.21	
	b. Persuade targeted stakeholders	i. No	−1.88 (0.22)	0	32.25
		ii. Yes	0	2.90	
	c. Persuade peripheral stakeholders	i. No	−1.10 (0.15)	0	18.89
		ii. Yes	0	1.70	
	d. Inform stakeholders about key issues	i. No	−1.42 (0.18)	0	24.26
		ii. Yes	0	2.18	
4. Imposed research use	a. Organisation mandates research use	i. No	−3.37 (0.60)	0	43.69
		ii. Yes	0	3.93	
	b. Organisation expects research use	i. No	−2.64 (0.44)	0	34.31
		ii. Yes	0	3.09	
	c. Organisation encourages research use	i. No	−1.70 (0.39)	0	22.00
		ii. Yes	0	1.98	

^a^All *P <*0.001 (using Wald χ^2^ statistics).

^b^Utility coefficients were rescaled so that they are positive, that the lowest level of each subaction has a zero-coefficient, and add up to 9.

**Figure 4:**
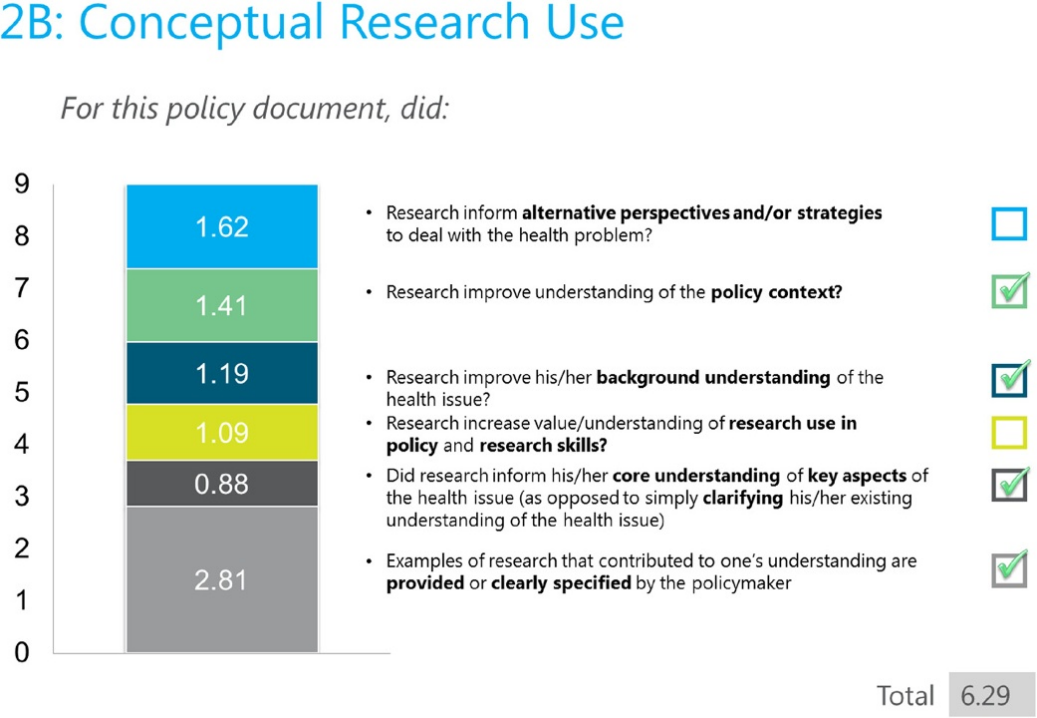
Scoring tool for conceptual research use.

Using conceptual research use as an example, if it was evident that research increased the policymakers’ core understanding (utility = 0.88) of background aspects of the health issue (utility = 1.19) and the policy context (utility = 1.41), and he/she cited specific examples of research (utility = 2.81), he/she would be assigned a score of 0.88 + 1.19 + 1.41 + 2.81 = 6.29 (out of 9), which would represent moderate conceptual research use (Figure 4)*.* The full scoring tool is provided in Additional file 3.

## Discussion

We have used conjoint analysis with a sample of experts with experience in health policy and research to develop the first empirically derived system of scoring research use in policy development. Conjoint analysis provided a systematic and innovative method of quantifying the relative importance of subactions for each research use domain measured in SAGE. To our knowledge, the current study represents the first attempt to numerically quantify the relative importance of different research use subactions. The consistency of the present findings to previous qualitative research points to the face validity and appropriateness of our scoring system.

### Summary and exploration of findings in relation to previous research

Beginning with conceptual use, the subaction with the greatest importance was whether policymakers could specify examples of research that contributed to their understanding of the policy issue (subaction 1f). This finding is in contrast with previously published views of knowledge translation researchers and the findings of qualitative research conducted among health decision makers, which suggest that conceptual research use is often an indirect, diffuse, and gradual process by which research shapes ideas and beliefs that subsequently influence policy [19,46,70-72]. Our results suggest that experts regard the ability to retrieve specific research as representing a stronger form of conceptual use.

In terms of specific types of conceptual understanding, the subaction with the greatest importance to conceptual use was whether research increased understanding of alternative strategies and perspectives to deal with the health issue (subaction 1d). In a number of qualitative interview studies, policymakers have stated that they primarily use research to identify new approaches to deal with current problems [40,72,73], and to determine the advantages and disadvantages of these options. It is this knowledge that eventually informs the content and direction of future policies. The next highest subaction was where research increased understanding of the policy context (e.g. the target population, neglected issues, priorities and needs, targets for future action; subaction 1b). Indeed, numerous qualitative interview studies with decision makers in a range of health areas suggest that research is used often by policymakers to gain a greater understanding of the characteristics, needs, and preferences of potential service users [3,70], broader policy issues [70], neglected health issues, and important targets for future action [43,72].

For instrumental use, the subaction with the greatest importance was the extent to which research directly influenced the policy document’s content. From the rescaled utilities, ratings of instrumental use were most influenced by whether policymakers used research to directly inform the core components of a decision, view, or course of action (subaction 2a.i). The utility for this subaction level was more than double that of using research to provide additional details to inform a decision or course of action (subaction 2a.ii). This result is unsurprising given that previous studies examining research use among health decision makers indicate that true instrumental use is where research has direct and concrete impacts on the formulation, implementation, and evaluation of policies, programs, and services [5,19,46,74], rather than just refining or supplementing predetermined decisions [43]. Lavis et al. [5] also described that instrumental research use occurs when policy documents explicitly cite research. Our findings agree with this, as the utility for subaction 2b (i.e. the policymaker clearly specified examples of research that contributed to the decision) had a significant and non-trivial impact on respondents’ ratings of instrumental use.

For tactical use, the subaction with the greatest importance was where research was used to provide evidence to influence targeted (as opposed to peripheral) stakeholders to support or act upon an established decision or view (subaction 3b). In support of these findings, evidence suggests that the primary tactical use of research is to provide ammunition for a decision or course of action so that stakeholders will support the existing decision [47,75], provide funding for the decision [3,43], contribute to the implementation of policies [16,76], or delay making decisions on particular issues [47]. Furthermore, research by El-Jardali et al. [7] indicated that, in developing countries, gaining stakeholder support is essential to improving the general climate towards research use among policymakers and enabling evidence-informed policy initiatives. It is well known that policymaking is highly influenced by a range of stakeholders, particularly those with power and political influence [76]. Therefore, it is not unusual that attempting to persuade stakeholders’ beliefs and actions through research emerged as the most important subaction of tactical use.

Using research to back up a predetermined action (subaction 3a) emerged as the next most important subaction of tactical use. This aligns with previous qualitative research demonstrating that policymakers often report using research as a form of reassurance to justify or confirm a predetermined decision to oneself and others [43]. Using research to inform stakeholders about the current status of the problem (subaction 4) obtained a similar importance value. Policymakers have reported using research to inform stakeholders about key aspects of health issues such as background details (e.g. rates, nature or complexity, medical, or technical issues) [3,43], neglected areas of need [43], alternative options, preferences of users [70], and priorities for future action [72]. It is interesting, however, that merely informing stakeholders emerged as a comparably important subaction of tactical use for our respondents. Respondents may have perceived that using research to inform stakeholders about a particular health issue will likely encourage them to support and act upon policies relating to that issue.

Finally, respondents’ ratings of imposed research use were most strongly influenced by organisations mandating (subaction 4a), as opposed to expecting (subaction 4b) or encouraging (subaction 4c), research use among staff. Indeed, previous research suggests that when organisations impose research use, they formally require or mandate staff to use research in policy by implementing strict policymaking guidelines, have compulsory knowledge translation programs and workshops, and apply performance management systems that incorporate research use and skills in retention and promotion decisions [77-80].

### Advantages of SAGE

In the introduction, we summarised the numerous benefits of developing measures of research use, and the limitations of previous measures. SAGE has been developed to overcome many of these limitations [9,36]. For example, SAGE is strongly based on a theoretical model, the SPIRIT Action Framework [28], and comprehensively assesses the different ways that research can be used in the development of a specific policy document.

Another key advantage of SAGE is that it incorporates the combination of a structured, qualitative interview and analysis of a corresponding policy document. The policy document can be used to identify explicit references to research, thus providing an objective means of assessing instrumental research use. Interviews, on the other hand, can unravel the broad application of research in policy development such as the more diffuse forms of research use (e.g. conceptual, tactical), the influence of research on agenda-setting or other priority setting exercises surrounding the policy [45], the contextual factors, capacity, barriers, and facilitators underlying research use, and the research engagement actions undertaken to obtain research. As a result, interviews can help to unravel the complex ways policymakers use research to inform policy or program development [81]. The use of a combination of measures provides an integrated, holistic, and valid approach to assessing research use in health policy [9].

The scoring system described in the current paper is one of the major advantages of SAGE over previous measures of research use in a discrete policy document (e.g. [37]). Not only have we developed a measure that separates each research use domain into its key subactions, but we have used conjoint analysis for the first time to calculate utilities and scores that quantify the relative importance of different research use subactions, based on the opinions and preferences of experts in health policy and research.

We considered using qualitative methods of obtaining expert opinion such as verification and Delphi panels [82-85]. However, these approaches do not provide a systematic means of assigning numeric scores to individual subactions. Conjoint analysis, on the other hand, provides a systematic statistical method of assigning utilities (i.e. scores) to each subaction, thus enabling the calculation of total scores for each research use domain. Because of these advantages, conjoint analysis was used in the present study.

Obtaining these utilities and importance values has two primary advantages. Firstly, it allows SAGE users to calculate appropriate, face-valid scores for each research use domain. Secondly, policy organisations using SAGE can identify the most important components of each research use domain, and use this information to invest in capacity-building interventions to improve subsequent research use. For example, the most important conceptual research use subaction (besides retrieving specific examples of research) was whether research informed policymakers’ understanding of alternative perspectives and strategies to deal with the health issue. Such conceptual understanding is important, as it may encourage policymakers to question assumptions and current practices, generate alternative strategies and recommendations, and influence what courses of action should or should not be adopted [43]. Using this information, policy organisations can encourage policymakers to focus on the actionable components of research, and to invest in programs and resources to help staff use research effectively so they can identify and comprehend alternative strategies, and adapt these to the current policy context [22,80]. SAGE can then be used to assess whether or not these capacity building interventions were effective in improving research use in policy. Along these lines, SAGE is currently being used as the main outcome measure in SPIRIT, a longitudinal study examining the impact of a multifaceted program on the capacity of health organisations to use research in the development of policies and programs (see [86] for details).

### Limitations, theoretical issues, and ideas for future research

It is possible that the subactions included in the conjoint analysis (and thus, in the scoring tool) did not capture the complete breadth of each research use domain. However, given that we conducted extensive qualitative analyses of over 65 interviews with policymakers and extensively reviewed previous literature on evidence-informed health policymaking, we are confident that the main components of each research use domain were included.

Although the SAGE scoring tool quantifies multiple subactions of research use, it does not quantify in-depth issues such as why research was used and the context in which it is used. Importantly, however, these aspects are addressed in the SAGE interview (Additional file 1). We believe that these aspects should be explored qualitatively due to their complexity. These qualitative details can be used to contextualise and add explanatory understanding to the scores obtained with the SAGE scoring tool.

The number of subactions for each research use domain did not exceed six. We did this to ensure the conjoint survey was not too onerous to complete. However, we must note that participants had already completed six other conjoint surveys [48] by the time they reached the first research use survey. Therefore, there is a possibility that they may have been mentally fatigued while doing the research use surveys, thereby reducing their ability to discriminate between profiles [50]. However, we are reassured by the fact that the relative importance of each research use subaction was, for the most part, consistent with previous research. Furthermore, only one respondent exhibited reversals in two of the surveys, and no respondents gave identical ratings for all profiles. This suggests that our sample was engaged and were able to discriminate between profiles, thus supporting the validity of our findings.

There are limitations relating to the interview-based nature of SAGE. For example, the interview only targets aspects of research use that policymakers can consciously retrieve. Qualitative research among mental health decision makers indicates that research can also shape policy makers’ understanding of policy issues gradually, indirectly, and unconsciously [40,46]. These unconscious (conceptual) aspects of research use are not directly measured in SAGE, although arguably such measurement would be very hard to do so in practice. Oliver et al. [87] have also argued that interviews often impose a dichotomy between research and policymaking and thus do not accurately reflect how policymakers use research in practice. Instead, they claim that observational studies provide a more valid method of evaluating policymakers’ use of research, and that such research is lacking. Although SAGE is interview-based, it goes some way towards addressing these issues by incorporating analysis of an actual policy document. We agree that direct observation of policymakers would provide the most effective way of assessing policymakers’ use of research, but there are likely to be significant feasibility issues due to the probable inconvenience to policy agencies of having an observer, as well as issues relating to privacy and confidentiality.

The sample size for our study was relatively small compared to other conjoint analysis studies and sample size recommendations [69,88]. However, large sample sizes are only possible if the target population is large [69, 88], which is not the case here. Orme [69] recommended that for investigational work and developing hypotheses about a particular group, 30 to 60 respondents may be sufficient, and our findings are consistent with these guidelines. Nevertheless, future studies may benefit from employing a larger sample size in order to verify the reliability of the utilities and importance values obtained in the present study. Future research must also incorporate a more ethnically diverse sample, including experts from developing countries, as they may have different perspectives regarding the relative importance of each research use subaction. Until such research is undertaken, it is uncertain as to whether the scoring tool can be used to assess research use in developing countries.

A final key issue relates to the generalisability of the SAGE scoring tool. Specifically, the utilities (scores) obtained from the conjoint analysis represent the relative importance of subactions in the context of policymaking in general. It is possible that these utilities do not apply to all kinds of policies (e.g., treatment guidelines, models of care, service delivery arrangements), and that different utilities would have emerged if a particular policy type or issue was specified. Further validity testing of SAGE is required to determine whether it can be used to measure research use across a broad range of policies and contexts.

## Conclusions

In this study, we have used conjoint analysis to develop an innovative system to score four types of research use in policy development. The scoring system is based on experts’ opinions regarding which subactions are most representative of each type of research use. This novel method has allowed us to establish a context-sensitive scoring system that will allow policy organisations to effectively quantify their level of research use, help them determine the critical components of each research use domain, and trigger investment in programs and resources to improve subsequent research use capacity within the organisation.

## Endnote

^1^In a typical conjoint analysis, subactions would be referred to as attributes [56]; however, we used the term subactions to enhance clarity, comprehension, and consistency of terms throughout this paper.

## Additional files

Additional file 1: **SAGE: Assessing the use of research in policy products - Interviewer’s guide.** (PDF 249 kb)
Additional file 2: **Profiles and questions in the SAGE ONLINE survey.** (PDF 58 kb)
Additional file 3: **SAGE scoring tool.** (PDF 383 kb)

## Abbreviations

CIPHER: Centre for Informing Policy in Health with Evidence from Research
OMEP: Orthogonal Main Effects Plan
SAGE: Staff Assessment of enGagement with Evidence from research
SPIRIT: Supporting Policy in Health with Research: an Intervention Trial
