# Supplementary material for: Using conjoint analysis to develop a system of scoring policymakers’ use of research in policy and program development
Source: Health Res Policy Syst. 2015 Aug 4;13:35. doi: 10.1186/s12961-015-0022-y (PMC4523001; doi:10.1186/s12961-015-0022-y)
Supplement: Additional file 2: — Profiles and questions in the SAGE ONLINE survey. (PDF 58 kb) [file 12961_2015_22_MOESM2_ESM.pdf]

Profiles and questions in the SAGE ONLINE survey

1. Conceptual Research Use

Listed below are eight different scenarios reflecting how a policymaker used research to provide new ideas, understanding, or concepts to influence his/her thinking about the current policy question.

Please rate, on the 1-9 scale below, the **extent to which each scenario represents a *limited, moderate, or extensive* form of conceptual research use**.

1	2	3	4	5	6	7	8	9
Limited			Moderate			Extensive		

Profile	Rating out of 9
1. Research:<br>- Improved understanding of the policy context (e.g., current health concerns and priorities).<br>- Informed alternative perspectives and/or strategies to deal with the health problem<br>- Informed one's core understanding of key aspects of the health issue<br>• Examples of such research are clearly specified or provided	
2. Research:<br>- Improved background understanding of the health issue<br>- Increased understanding of research use in policy and research skills<br>- Informed one's core understanding of key aspects of the health issue<br>• Examples of such research are clearly specified or provided	
3. Research:<br>- Increased understanding of research use in policy and research skills<br>- Informed alternative perspectives and/or strategies to deal with the health problem<br>- Clarified one's existing understanding of the health issue<br>• Examples of such research are clearly specified or provided	
4. Research:<br>- Improved background understanding of the health issue<br>- Improved understanding of the policy context (e.g., current health concerns and priorities).<br>- Clarified one's existing understanding of the health issue<br>• Examples of such research are clearly specified or provided	
5. Research:<br>- Improved background understanding of the health issue<br>- Informed alternative perspectives and/or strategies to deal with the health problem<br>- Informed one's core understanding of key aspects of the health issue<br>• Examples of such research are NOT clearly specified or provided	
6. Research:<br>- Improved understanding of the policy context (e.g., current health concerns and priorities).<br>- Increased understanding of research use in policy and research skills	

- Informed one's core understanding of key aspects of the health issue<br>• Examples of such research are NOT clearly specified or provided	
7. Research:<br>- Improved background understanding of the health issue<br>- Improved understanding of the policy context (e.g., current health concerns and priorities).<br>- Increased understanding of research use in policy and research skills<br>- Informed alternative perspectives and/or strategies to deal with the health problem<br>- Clarified one's existing understanding of the health issue<br>• Examples of such research are NOT clearly specified or provided	
8. Research clarified one's existing understanding of the health issue<br>• Examples of such research are NOT clearly specified or provided	

2. Instrumental Research Use

Listed below are eight different scenarios reflecting how a policymaker used research to develop the content or direction of the current policy.

Please rate, on the 1-9 scale below, the **extent to which each scenario represents a *limited, moderate, or extensive* form of instrumental research use**.

1	2	3	4	5	6	7	8	9
Limited			Moderate			Extensive		

Profile	Rating out of 9
1.<br>• Research directly influenced the core of a decision, view, or course of action<br>• Examples of such research are clearly specified or provided	
2.<br>• Research refined/informed peripheral additional details of a decision or course of action<br>• Examples of such research are clearly specified or provided	
3.<br>• Research had a vague and negligible role in shaping the decision or course of action<br>• Examples of such research are clearly specified or provided	
4.<br>• Research directly influenced the core of a decision, view, or course of action<br>• However, examples of such research are NOT clearly specified or provided	
5.<br>• Research refined/informed peripheral additional details of a decision, view, or course of action<br>• However, examples of such research are NOT clearly specified or provided	
6.<br>• Research had a vague and negligible role in shaping the decision or course of action<br>• However, examples of such research are NOT clearly specified or provided	

3. Tactical Research Use

Listed below are eight different scenarios reflecting how a policymaker used research to justify or lend weight to pre-existing preferences and actions.
Please rate, on the 1-9 scale below, the **extent to which each scenario represents a *limited, moderate, or extensive* form of tactical research use**.

1	2	3	4	5	6	7	8	9
Limited			Moderate			Extensive		

Profile	Rating out of 9
1. The policymaker used research to:<br>• Support, confirm, or justify established positions or decisions relating to the issue<br>• Inform stakeholders about key issues relating to the health issue<br>• Provide hard evidence to persuade the following stakeholders to support an existing decision or view:<br>- peripheral stakeholders (e.g., professional bodies, civil society organisations, interest groups)	
2. The policymaker used research to:<br>• Inform stakeholders about key issues relating to the health issue<br>• Provide hard evidence to persuade the following stakeholders to support an existing decision or view:<br>- peripheral stakeholders (e.g., professional bodies, civil society organisations, interest groups)	
3. The policymaker used research to:<br>• Support, confirm, or justify established positions or decisions relating to the issue<br>• Inform stakeholders about key issues relating to the health issue<br>• Provide hard evidence to persuade the following stakeholders to support an existing decision or view:<br>- Primary/targeted stakeholders (e.g., consumers, service providers, politicians, staff)	
4. The policymaker used research to:<br>• Inform stakeholders about key issues relating to the health issue<br>• Provide hard evidence to persuade the following stakeholders to support an existing decision or view:<br>- Primary/targeted stakeholders (e.g., consumers, service providers, politicians, staff)	
5. The policymaker used research to:<br>• Support, confirm, or justify established positions or decisions relating to the issue<br>• Provide hard evidence to persuade the following stakeholders to support an existing decision or view:<br>- primary/targeted stakeholders (e.g., consumers, service providers, politicians, staff)<br>- peripheral stakeholders (e.g., professional bodies, civil society	

organisations, interest groups)	
6. The policymaker used research to:<br>• Provide hard evidence to persuade the following stakeholders to support an existing decision or view:<br>- primary/targeted stakeholders (e.g., consumers, service providers, politicians, staff)<br>- peripheral stakeholders (e.g., professional bodies, civil society organisations, interest groups)	
7. The policymaker used research to:<br>• Support, confirm, or justify established positions or decisions relating to the issue	
8. The policymaker:<br>• Did not refer to any specific tactical forms of research use	

4. Imposed Research Use

Listed below are four different scenarios reflecting the degree to which organisational pressures influenced the policymaker's use of research in developing the current policy document

Please rate, on the 1-9 scale below, the **extent to which each scenario represents a *limited, moderate, or extensive* form of imposed research use**.

1	2	3	4	5	6	7	8	9
Limited			Moderate			Extensive		

Profile	Rating out of 9
1. The organisation...<br>• Expected research to be used<br>• Encouraged research use	
2. The organisation...<br>• Mandated research use<br>• Encouraged research use	
3. The organisation...<br>• Mandated research use<br>• Expected research to be used	
4. The organisation...<br>• Neither Encouraged, Expected, nor Mandated research use	
