# Supplementary material for: Using conjoint analysis to develop a system of scoring policymakers’ use of research in policy and program development
Source: Health Res Policy Syst. 2015 Aug 4;13:35. doi: 10.1186/s12961-015-0022-y (PMC4523001; doi:10.1186/s12961-015-0022-y)
Supplement: Additional file 3: — SAGE scoring tool. (PDF 383 kb) [file 12961_2015_22_MOESM3_ESM.pdf]

SAGE scoring tool

© The Sax Institute

Instructions

1. This scoring guide requires you to 4 types of research use in relation to a policy document
2. Each construct is listed on a separate page. Each page is equivalent to a checklist
   - Each item on the checklist refers to a behaviour or action
3. You will use the checklist to score policymakers’ responses to an interview (i.e., the SAGE interview) about a specific policy document. You will also have the policy document itself to help you score.
   - The interview is divided into sections corresponding to the four research use domains in this scoring guide
4. How to use this scoring guide with the interview
   - Read the policymakers’ responses to a particular section (e.g., “*conceptual use*”)
   - Go to the page in this scoring guide corresponding to that section (e.g., “II: *Conceptual use”, p*.5)
   - If the policy maker has performed a particular action, based on their interview response in this section, tick it off on the checklist
   - Please use the **definitions of terms** following each section to help you determine whether the policymaker has performed this action or not. The glossary contains detailed definitions for all terms highlighted in bold within the scoring guide.
5. Add up the scores displayed for each ticked action to obtain a total score for that particular construct

© The Sax Institute

I: Instrumental Research Use

saxinstitute

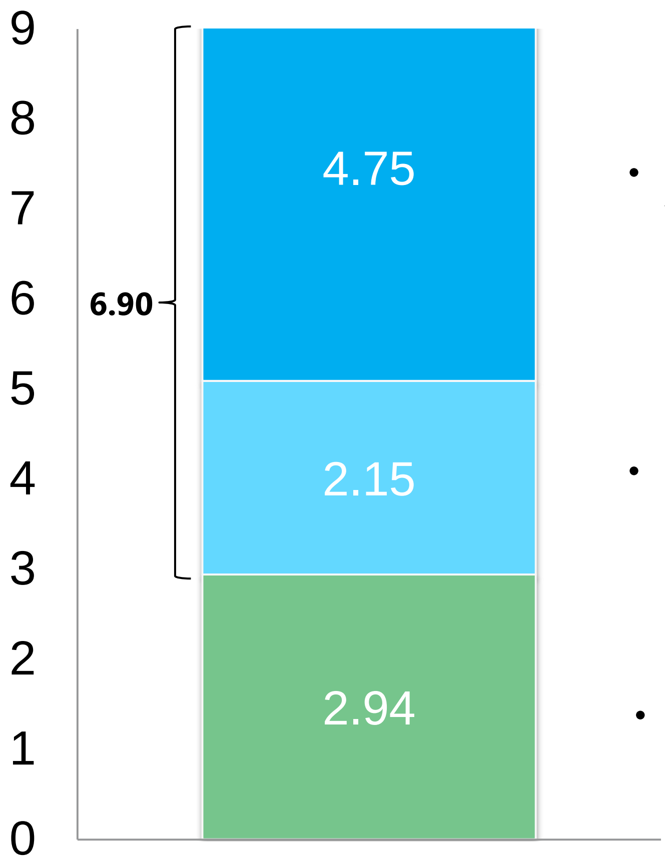

- Did research directly inform the **core or majority of the decision, view,** or **course of action,** relating to this policy document?*

OR

- Did research merely **inform peripheral or additional details** of the decision, view, or course of action, relating to this policy document

- Examples of research that influenced the decision, view, or course of action are **clearly specified** or **provided**

*If this behaviour is ticked, score 4.75+2.15=6.90
If research had a **distant or vague influence**, or **no influence at all**, on the decision, view, or course of action relating to this policy document, score 0 in this category

Total

© The Sax Institute

I: Instrumental Use definitions

INSTRUMENTAL USE OF RESEARCH

Use of research to directly develop content or direction of policy/programs

INSTRUMENTAL RESEARCH USE IN AGENDA SETTING

Use of research to help identify which issues or problems are priorities and should be addressed by policy/program development

INSTRUMENTAL RESEARCH USE IN POLICY DEVELOPMENT

Use of research to help determine the direction and content of policies/programs and policy/program documents

CLEARLY SPECIFIED: The policymaker provides specific examples of research that contributed to the decision, view, or course of action described in the policy document (e.g., he or she may mention a particular systematic review, primary research paper, internal policy document, analysed dataset etc.) OR the policy document itself cites specific research sources when describing the decision, view, or course of action.

CORE OF A DECISION: Research directly influenced the main aspects of a particular course of action [or inaction], recommendation, decision, or action plan. The essence of the decision or course of action was directly influenced by research. Research was the primary influence on the course of action.

PERIPHERAL ADDITIONAL DETAILS: Research informed additional details, elements, or aspects of an existing decision/course of action. The core of the decision/course of action was primarily influenced by factors other than research. Consequently, research contributed to additional elements, as opposed to the core of the decision or course of action. Research was used to further refine the course of action.

VAGUE, NEGLIGIBLE ROLE: Research had a minimal, negligible influence on the development of the policy document. This is evident from the policy maker, who claims that research only played a small role in the development of the document (potentially because the policy document was influenced primarily by other factors). This may also be evident from the policy document itself, if the policy exhibits minimal reference to research, is used sparingly in the document, or most of the policy's contents does not appear to be based on research

© The Sax Institute

II: Conceptual Research Use

For this policy document, did:

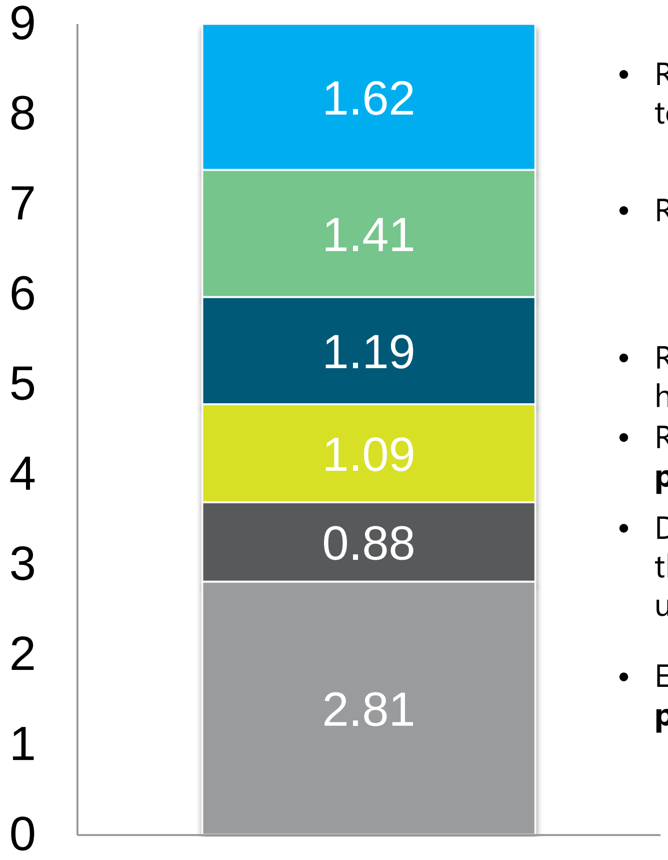

- Research inform **alternative perspectives and/or strategies** to deal with the health problem?
- Research improve understanding of the **policy context?**
- Research improve his/her **background understanding** of the health issue?
- Research increase value/understanding of **research use in policy** and **research skills?**
- Did research inform his/her **core understanding** of **key aspects** of the health issue (as opposed to simply **clarifying** his/her existing understanding of the health issue)
- Examples of research that contributed to one's understanding are **provided** or **clearly specified** by the policymaker

Total

© The Sax Institute

II: Conceptual Use definitions

CONCEPTUAL USE OF RESEARCH

Use of research to provide new ideas, understanding or concepts that influence thinking about policy/programs

ALTERNATIVE IDEAS, STRATEGIES, PERSPECTIVES:

Research shed light on alternative ideas or helped challenge pre-existing ideas, for example:

a) New and promising strategies, approaches, ideas, service models, models of care etc.

b) Research ideas

c) Revaluation of current services, health systems, priorities, or institutional arrangements

d) Revaluation of attitudes to the health problem

BACKGROUND UNDERSTANDING OF HEALTH ISSUE: This includes a greater understanding of:

a) Background, causes, risk factors, current statistics or rates

b) Associations or links between various health phenomena

c) Nature and/or complexity of the health problem

CLEARLY SPECIFIED: The policymaker provides specific examples of research that contributed to his/her conceptual understanding or thinking about the current issue

ESSENTIAL OR CORE: Research informed the policy makers' core understanding of the health issue. Research provided an essential understanding of the issue and/or the essential, important, or focal components of the issue. Research contributed to one's core understanding of the health issue and it's key aspects or elements

© The Sax Institute

II: Conceptual Use definitions cont'd

EXISTING UNDERSTANDING: Research clarified what the policy maker already knew about the health issue OR research built upon one's general, existing understanding of the issue, refining knowledge of additional detials of the health issue, rather than providing new insights into important or basic aspects of the health issue.

POLICY CONTEXT, CURRENT HEALTH CONCERNS, AND PRIORITIES:

A greater understanding of:

a) Aspects of the target population (e.g., cultural norms, values, needs, socioeconomic factors, minority or gender issues)

b) Problems with and gaps in current services

c) Interconnections between various social phenomena

d) Current health needs, neglected issues, current health priorities/targets for action, health goals

RESEARCH USE IN POLICY AND RESEARCH SKILLS:

a) Research skills to facilitate policy development (including professional development and continuing education to maintain expertise)

b) Knowledge and attitudes towards research use in policy development

© The Sax Institute

III: Tactical Research Use

saxinstitute

Did the policymaker use research to:

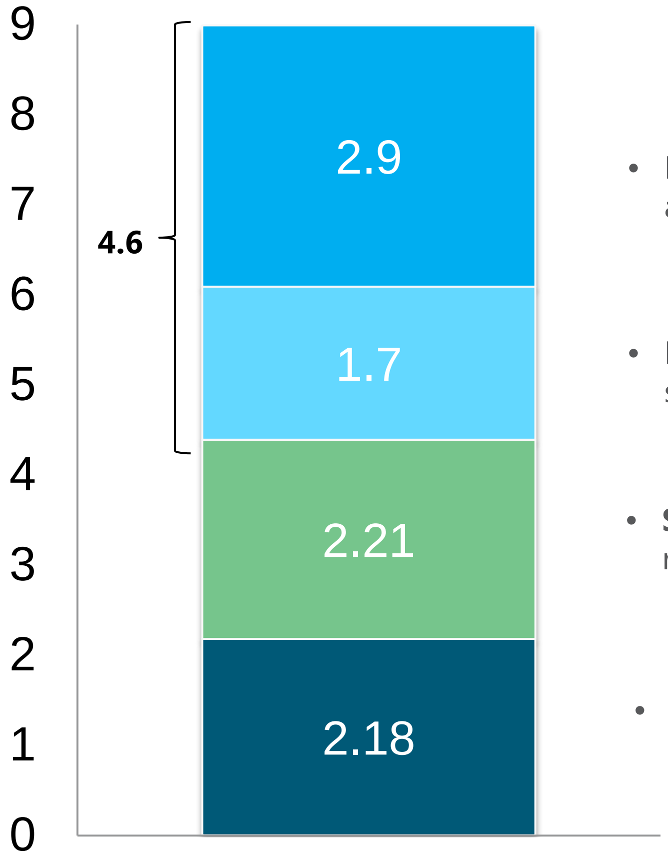

- Provide hard evidence to **persuade *targeted stakeholders*** to support an existing decision or view?‡ ☐
- Provide hard evidence to **persuade *peripheral stakeholders*** to support an existing decision or view? ☐
- **Support, confirm,** or **justify** established positions or decisions relating to the issue? ☐
- **Inform stakeholders** about **key issues** relating to the health issue? ☐

‡If this category is ticked, score 2.9+1.7=4.6

Total

© The Sax Institute

III: Tactical Use definitions

TACTICAL/SYMBOLIC USE OF RESEARCH: Use of research to justify or lend weight to pre-existing preferences and actions

KEY ISSUES OF THE HEALTH PROBLEM: Key issues include: the health needs of the target population, priorities, statistics and background to the health issue, possible strategies, and additional conceptual aspects

PERIPHERAL/ADDITIONAL STAKEHOLDERS: Additional stakeholder groups that aren't directly being affected by the policy, but have an interest in the issue or policy. These include: Interest groups such as sectional groups (e.g., trade unions, employers' associations, bodies representing the professions – producer interests); civil society organisations (e.g., non-government and community-based organisations such as non-health entities, sporting clubs, schools, unions, special interest groups, Aboriginal and Torres Strait Islander Groups; cultural and faith-based organisations) and cause groups (e.g., campaigning groups, typically representing the interests of consumers, such as those on abortion, human rights, the environment); the media, industry, or corporations (e.g., pharmaceutical companies, alcohol and tobacco industry)

STAKEHOLDERS: Individuals and groups with an interest in an issue or policy, those that are affected by a policy, and those who play a role in relation to making or implementing the policy (also known as actors).

© The Sax Institute

saxinstitute

III: Tactical Use definitions cont'd

TACTICAL/SYMBOLIC USE OF RESEARCH: Use of research to justify or lend weight to pre-existing preferences and actions

KEY ISSUES OF THE HEALTH PROBLEM: Key issues include: the health needs of the target population, priorities, statistics and background to the health issue, possible strategies, and additional conceptual aspects

PERIPHERAL/ADDITIONAL STAKEHOLDERS: Additional stakeholder groups that aren't directly being affected by the policy, but have an interest in the issue or policy. These include: Interest groups such as sectional groups (e.g., trade unions, employers' associations, bodies representing the professions – producer interests); civil society organisations (e.g., non-government and community-based organisations such as non-health entities, sporting clubs, schools, unions, special interest groups, Aboriginal and Torres Strait Islander Groups; cultural and faith-based organisations) and cause groups (e.g., campaigning groups, typically representing the interests of consumers, such as those on abortion, human rights, the environment); the media, industry, or corporations (e.g., pharmaceutical companies, alcohol and tobacco industry)

STAKEHOLDERS: Individuals and groups with an interest in an issue or policy, those that are affected by a policy, and those who play a role in relation to making or implementing the policy (also known as actors).

© The Sax Institute

IV: Imposed Research use

Did the organisation:

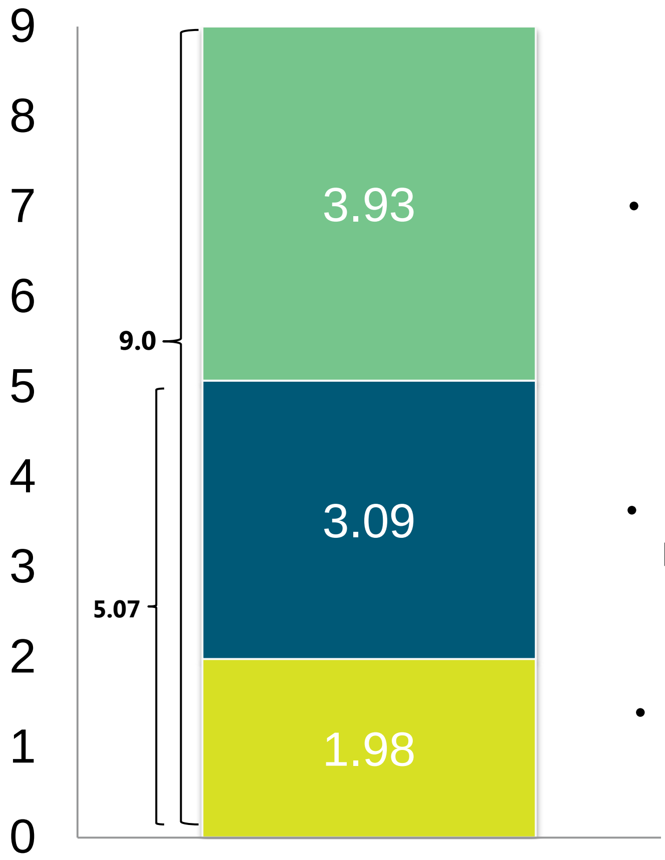

- **Mandate** research use?†
- **Expect** research use and/or **express research use as best practice**?‡
- **Encourage** research to be used?

†If the organisation mandates research use, score a 9 in this category
‡If the organisation expects research use, score 3.09+1.98=5.07 in this category

Total

© The Sax Institute

saxinstitute

IV: Imposed Research use definitions

IMPOSED USE OF RESEARCH

Use of research to meet organizational, legislative or funding requirements that research be used.

ENCOURAGED: Research use is actively supported and/or fostered by the organisation, although non-use of research is not frowned upon. For example, document templates do not have clear sections where research can be incorporated and/or it may be signed off even if research is not used.

EXPECTED: Research use is expected, or assumed by the organisation (as opposed to explicitly demanded). Research use is viewed as the best practice by the organisation. For example, document templates have clear sections where research can be incorporated and/or documents are more likely to be signed off and distributed if research is used.

MANDATED: Research use is required, commanded, and/or demanded by the organisation. Research use is compulsory. For example, document templates have clear sections where research can be incorporated. Further, documents will not be signed off unless research is integrated.

© The Sax Institute
